# Osteoinduction Evaluation of Fluorinated Hydroxyapatite and Tantalum Composite Coatings on Magnesium Alloys

**DOI:** 10.3389/fchem.2021.727356

**Published:** 2021-09-07

**Authors:** Zheng Cao, Li Li, Linjun Yang, LiLi Yao, Haiyan Wang, Xiaoyang Yu, Xinkun Shen, Litao Yao, Gang Wu

**Affiliations:** ^1^ Department of Dentistry, Sir Run Run Shaw Hospital, School of Medicine, Zhejiang University, Hangzhou, China; ^2^ Department of Oral and Maxillofacial Surgery/Pathology, Amsterdam UMC and Academic Center for Dentistry Amsterdam (ACTA), Amsterdam Movement Science (AMS), Vrije Universiteit Amsterdam (VU), Amsterdam, Netherlands; ^3^ Department of Oral Implantology and Prosthetic Dentistry, Academic Centre for Dentistry Amsterdam (ACTA), University of Amsterdam (UvA) and Vrije Universiteit Amsterdam (VU), Amsterdam, Netherlands; ^4^ School and Hospital of Stomatology, Wenzhou Medical University, Wenzhou, China

**Keywords:** magnesium alloy, fluorinated hydroxyapatite, tantalum, osteogenesis, magnetron sputtering

## Abstract

Magnesium (Mg) alloys have a wide range of biomaterial applications, but their lack of biocompatibility and osteoinduction property impedes osteointegration. In order to enhance the bioactivity of Mg alloy, a composite coating of fluorinated hydroxyapatite (FHA) and tantalum (Ta) was first developed on the surface of the alloy through thermal synthesis and magnetron sputtering technologies in this study. The samples were characterized by scanning electron microscopy (SEM), atomic force microscopy (AFM), energy dispersive spectroscopy (EDS) mapping, X-ray diffraction (XRD), X-ray photoelectron spectroscopy (XPS), and water contact angle measurement (WCA), which characterized the surface alternation and confirmed the deposition of the target FHA/Ta coating. The results of cell morphology showed that the MC3T3-E1 cells on the surface of Mg/FHA/Ta samples had the largest spreading area and lamellipodia. Moreover, the FHA coating endowed the surface with superior cell viability and osteogenic properties, while Ta coating played a more important role in osteogenic differentiation. Therefore, the combination of FHA and Ta coatings could synergistically promote biological functions, thus providing a novel strategy for implant design.

## Introduction

Recently, numerous bone repair materials have been developed to replace autograft bones and are commercially available as bone substitutes. The currently available biodegradable implants usually comprise of bio-ceramics and resorbable polymers, with the poor mechanical strength of the bio-ceramics and polymers compromising their application (Upadhyay et al., 2020). Metallic materials are commonly used for repair or replacement of damaged bone tissue. Those currently widely employed in orthopedics include titanium alloys because of their good mechanical properties and cytocompatibility. However, the inert materials often need to be removed via invasive secondary surgeries once the bone has healed completely. Magnesium (Mg) and its alloys have attracted significant research attention as potential metallic implants and GBRs (guide osteogenesis membranes) due to exceptionally light weight, *in vivo* degradation, and excellent mechanical characteristics which can simulate natural bone (Zhang et al., 2015; Liu et al., 2016; Espiritu et al., 2021). In addition, Mg, an element essential for the metabolic process in the human body, primarily exists in bone tissue, stimulates bone cell proliferation, and enhances bone regeneration. Though Mg and its alloys have emerged as a new class of biodegradable metallic materials and can be widely used in orthopedic applications, the lack of biocompatibility and osteoinductive properties limits their widespread application (Chakraborty Banerjee et al., 2019). Therefore, owing to their poor anti-corrosion behavior and the lack of osteogenic properties, the bone and tissue regeneration around Mg alloy implants is observed to be non-optimal (Lin et al., 2013; Yang et al., 2015; Liu et al., 2017; Rahman et al., 2020). In this respect, the surface modification of Mg-based materials plays a pivotal role by improving cellular response without affecting the desirable mechanical properties and enhancing osteogenic properties.

Fluoridated hydroxyapatite (FHA) coating—with optimal biocompatibility, biodegradability, and osteogenic properties—has been investigated to modify the orthopedic implants (Wang et al., 2021). Most importantly, the FHA coating possesses a nanoneedle structure which can mimic collagen fibris (Cai et al., 2007; Uddin et al., 2015; Zhao et al., 2016; Shen et al., 2018). FHA originates from hydroxyapatite (HA), where OH- in the HA lattice is substituted with F- to form FHA (Liu et al., 2019). In addition, FHA possesses excellent osteoconductivity and bioactivity. Compared with HA, FHA can be easily coated onto the biodegradable Mg substrates by electrochemical deposition. In addition, FHA is more stable, along with having a low dissolution, and high cell response (Ma et al., 2014; Wang et al., 2015; Wu et al., 2020). Therefore, the synthesis of FHA on the Mg implant surface represents a potential method to optimize osteogenic differentiation. In addition, tantalum (Ta) and its oxides have emerged as excellent materials, exhibiting biocompatibility, anti-corrosion performance, and osteoinductivity (Bencharit et al., 2014). Compared with the surface structure, the release of the Ta ions plays a pivotal role in osteogenic promotion, as has been confirmed in previous studies (Wang et al., 2015; Wu et al., 2020). Tantalum shows remarkable physicochemical stability and superior cytocompatibility, which have been confirmed can stimulate the proliferation and osteogenesis of osteoblasts (Bencharit et al., 2014). Further, the Ta nano-coating was fabricated by magnetron sputtering which is regarded as an efficient, predictable, and controllable method that is suitable for biomedical applications. Though the excellent biofunction has been proven, the combination of FHA coating and Ta nano-coating has not been investigated before.

In this study, the FHA and Ta coatings had been combined to modify the Mg alloys so as to improve their biocompatibility and osteogenic properties. It is suggested that the FHA coating constructed on the Mg implants endowed the surface with a bone bio-mimicking nano-needle structure, which could remarkably enhance osteogenic differentiation. The Ta coating on the FHA coating further enhanced the osteogenic properties. Further, it is speculated that the combination of the nano-needle structure and Ta ions function would synergistically enhance the osteogenic properties. Several issues are considered in this study: 1) the selection of the optimal sputtering parameter for the Ta coating to endow the surface with superior osteogenic properties, 2) investigating the function of FHA and Ta modified Mg surfaces, and 3) discussing the synergetic effect of nano-needle structure and Ta on osteogenic differentiation.

## Materials and Methods

### Preparation of Coating Samples

Round Mg alloy substrates (AZ31B) with a diameter of 15 mm were used for the experiments. The specimens were first gradually ground by using silicon carbide papers of 400, 600, 1,000, and 2000 grit, followed by washing with acetone, alcohol, and distilled water ultrasonically for 15 min each. The washed samples were stored in a vacuum to keep them clean.

Preparation of the FHA solution: the method for FHA solution was according to a previous study^[20]^. In short, 0.306 g calcium nitrate tetrahydrate [Ca (NO_3_)_2_ **·**4H_2_O] (AR, Aladdin, Shanghai, China), 0.100 g disodium hydrogen phosphate dodecahydrate [Na_2_HPO_4_ **.**12H_2_O] (AR, Aladdin, Shanghai, China), and 0.003 g sodium fluoride (NaF) (AR, Aladdin, Shanghai, China) were separately dissolved in 100 ml deionized water under magnetic stirring. Next, the Ca (NO_3_)_2_ solution was added dropwise to the disodium Na_2_HPO_4_ solution. Subsequently, the pH value of the mixed solution was adjusted to 6.80 by using nitric acid (HNO_3_) (denoted as HA solution). Afterward, 30 ml NaF solution was added dropwise to the HA solution, and the pH value of the mixed solution was adjusted to 6.30 by HNO_3_ (denoted as FHA solution).

Preparation of FHA coating: the prepared Mg foils were immersed in the FHA solution, and the coating solution was heated at a rate ranging between 40°C/min to 120°C/min, followed by boiling for 30 min. Afterward, the coated substrates were collected from the coating solutions, rinsed with distilled water, and air-dried. The as-obtained sample was labeled as Mg/FHA.

Preparation of Ta coating: Ta coating was deposited on the Mg foils and Mg/FHA by employing a DC magnetron sputtering machine (VTC-600-2HD, KeJing Auto-instrument Co., Shenyang, China). The magnesium foils and Mg/FHA were immobilized on a rotating sample stage. A Ta target of 99.9% pure Ta (ZhongNuo Advanced Material Technology Co., Beijing, China) was used during the experiment. The magnetron sputtering was performed using four different durations (1, 3, 5, and 7 min) so as to attain the optimal Ta coating. The fabricated samples were marked as Mg/Ta1, Mg/Ta3, Mg/Ta5, and Mg/Ta7, respectively. The optimal parameter duration (marked as Mg/FHA/Ta) was identified by cell viability and was applied in the subsequent magnetron sputtering steps.

### Coating Characterization

The surface morphology and topography of the different specimens were characterized by field emission scanning electron microscopy (FE-SEM, Nova 200 Nano SEM, America) equipped with energy dispersive spectroscopy (EDS, Zeiss AURIGA FIB, Germany) and atomic force microscopy (AFM, Bruker Dimension, Germany). The water contact angle of the different specimens was measured using a contact angle goniometer (WCA, SDC-200S, Sindin, China). The crystal structure of the specimens was characterized by X-ray diffraction (XRD, Smartlab-9kW). The surface chemical analysis of the samples was performed by X-ray photoelectron spectroscopy (XPS, Eden Prairie, MN).

### Cell Culture

Mouse embryo osteoblast precursor cells (MC3T3-E1, ATCC CRL-2594) were used to assess the *in vitro* biological function of the different samples. MC3T3-E1 cells were cultured in an α-Minimum Eagle’s Medium (α-MEM) (Gibco, ThermoFisher) supplemented with 10% fetal bovine serum (FBS) (Gibco, ThermoFisher) and 1% penicillin-streptomycin mixture (Beyotime, China). The cell passage was carried out until the cell confluence reached about 80–90%. During the cell culturing procedure, the culture medium was replaced every 2 days. The cell seeding was performed after the sterilized samples (diameter: 15 mm) were placed into the 24-well plates for all the *in vitro* experiments.

### Cell Morphology

The cell morphology was performed after seeding cells (1 × 10^4^ cells/well) on 24 well plates. The cell morphology was characterized by cell staining at precise time durations, according to the previous study (Wu et al., 2020). In order to better observe the cell morphology, the samples were placed after the cell seeding procedure. Thus, the cells are still in contact with the sample surfaces and the cell morphology could be clearly observed because of the smooth and flat plate. Fluorescence microscopy (FM) was employed to explore the cell morphology of the different samples. For this purpose, the cells were fixed by using 4% paraformaldehyde (Sigma, United States) for an hour after 2 days of cell culturing, followed by staining with Rhodamine labeled phalloidin (Beyotime, China) for an hour and DAPI (Beyotime, China) for 15 min, respectively. Subsequently, the cell morphology was detected by FM (Olympus IX71, Japan) after washing three times. Finally, the relative cell areas and lamellipodia areas were analyzed by using the ImageJ (1.51) software.

### Cell Viability

The MTT method was carried out to access the cell viability of different samples. Briefly, after the cell seeding on the 24 well plates with a concentration of 2 × 10^4^ cells/well, the incubated medium was replaced with 300 µL 10% MTT solution (Solarbio, China), which was diluted with α-MEM after four and 7 days of cell culturing. Next, 1 ml DMSO was used to replace the MTT solution after 4 hours of cell culturing, followed by shaking in a shaker for 10 min. Afterward, 200 µL solutions from each well were absorbed in 96-well plates (Corning Incorporated, United States). The microplate reader (BioRad 680, United States) was used for detection at a wavelength of 490 nm.

### Alkaline Phosphatase (ALP) Activity

The MC3T3-E1 cells were seeded on the substrates and cultured with α-MEM at a concentration of 2 × 10^4^ cells/well. After 24 h, the normal medium was replaced with the osteogenic induction medium (supplemented with 10 mmol/L β-glycerophosphate, 0.05 mmol/L acetic acid, and 100 mmol/L dexamethasone). The ALP assay kit (Jiancheng, Nanjing, China), as well as the BCA protein assay kit (Beyotime, China), were used to evaluate the ALP activity of MC3T3-E1 after cultivation for 4 and 7 days. The absorbance was subsequently measured at 405 nm wavelength by using a spectrophotometer, and the ALP activity was normalized with the total protein concentration.

### Mineralization Analysis

The MC3T3-E1 cells were seeded on the substrates and cultured with α-MEM at a concentration of 2 × 10^4^ cells/well. The culture medium was replaced with an osteogenic induction medium after 24 h. Afterward, the cells on the samples were fixed using 4% paraformaldehyde solution for 40 min, followed by staining with 40 nM Alizarin Red reagent (ARS, Solarbio, China) for 50 min after 7 and 14 days of osteogenic medium induction (six samples per group). Subsequently, the stained samples were washed with distilled water until the additional red color disappeared. Afterward, the dyed calcium nodules were dissolved in 10 wt% cetylpyridinium chloride solution (500 ml). Finally, 200 µL solution was absorbed in 96-well plates (Corning Incorporated, United States), followed by measurement using a Bio Rad microplate reader at 540 nm.

### Statistical Analysis

The data were expressed as the mean ± standard deviation (SD). The statistical analysis was performed by the one-way analysis of variance (ANOVA) by using the Fisher’s LSD multiple comparison test [confidence levels: 95% (*p* < 0.05), 99% (*p* < 0.01)].

## Results and Discussion

### The Selection of Optimal Tantalum Coating

**FIGURE 1:**
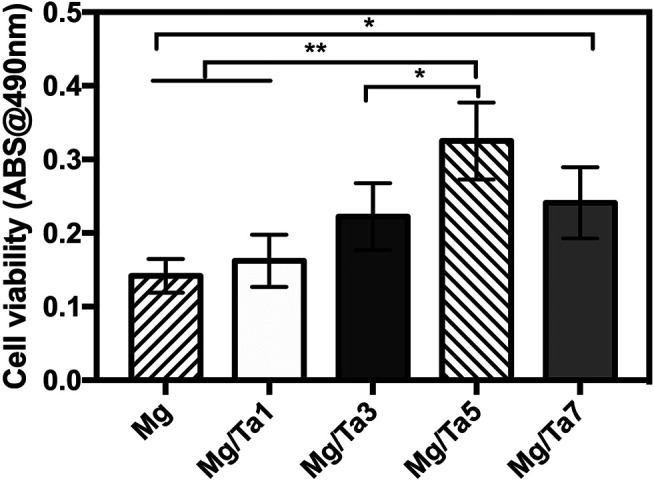
Cell viability of MC3T3-E1 cells on samples with different Ta sputtering durations (the data is displayed as mean ± standard deviation, *p < 0.05, **p < 0.01.) (*n* = 6).

The cell viability assay of the Mg substrates coated for different durations was used to optimize the magnetron sputtering parameter. As shown in Figure 1, the Mg substrates showed the lowest cell viability (ODs: 0.142 ± 0.023), which was mainly attributed to the Mg ion release leading to an increment in the pH value of the cell culture medium (Liu et al., 2018). Moreover, the Ta coated samples demonstrated increased cell viability as compared with the pure Mg substrates. For instance, Mg/Ta5 showed significantly increased cell viability as compared with the Mg substrates (ODs: 0.325 ± 0.052, *p* < 0.01). In addition, though Mg/Ta7 revealed superior cell viability compared to Mg (ODs: 0.241 ± 0.048, *p* < 0.05), however, the cell viability was decreased as compared with Mg/Ta5. Therefore, it could be concluded that Mg/Ta5 possessed the optimal cytocompatibility. The reason was mainly attributed to the optimal Ta release, which was consistent with a previous study (Bencharit et al., 2014). Therefore, the tantalum nanolayer (Mg/Ta5) possessed cell behavior promotion properties, which provide beneficial conditions for cell growth. Thus, it was employed in the subsequent experiments.

### Surface Characterization

**FIGURE 2:**
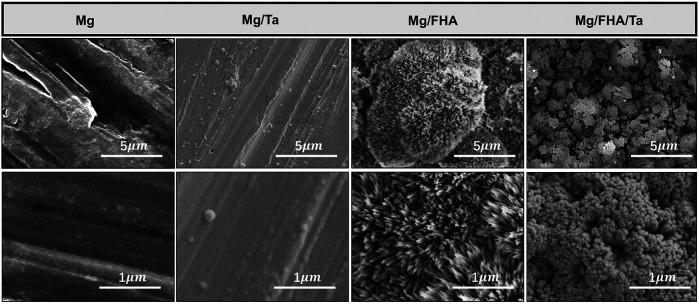
Top-view SEM images of different samples including high magnification and low magnification images (*n* = 6).

**FIGURE 3:**
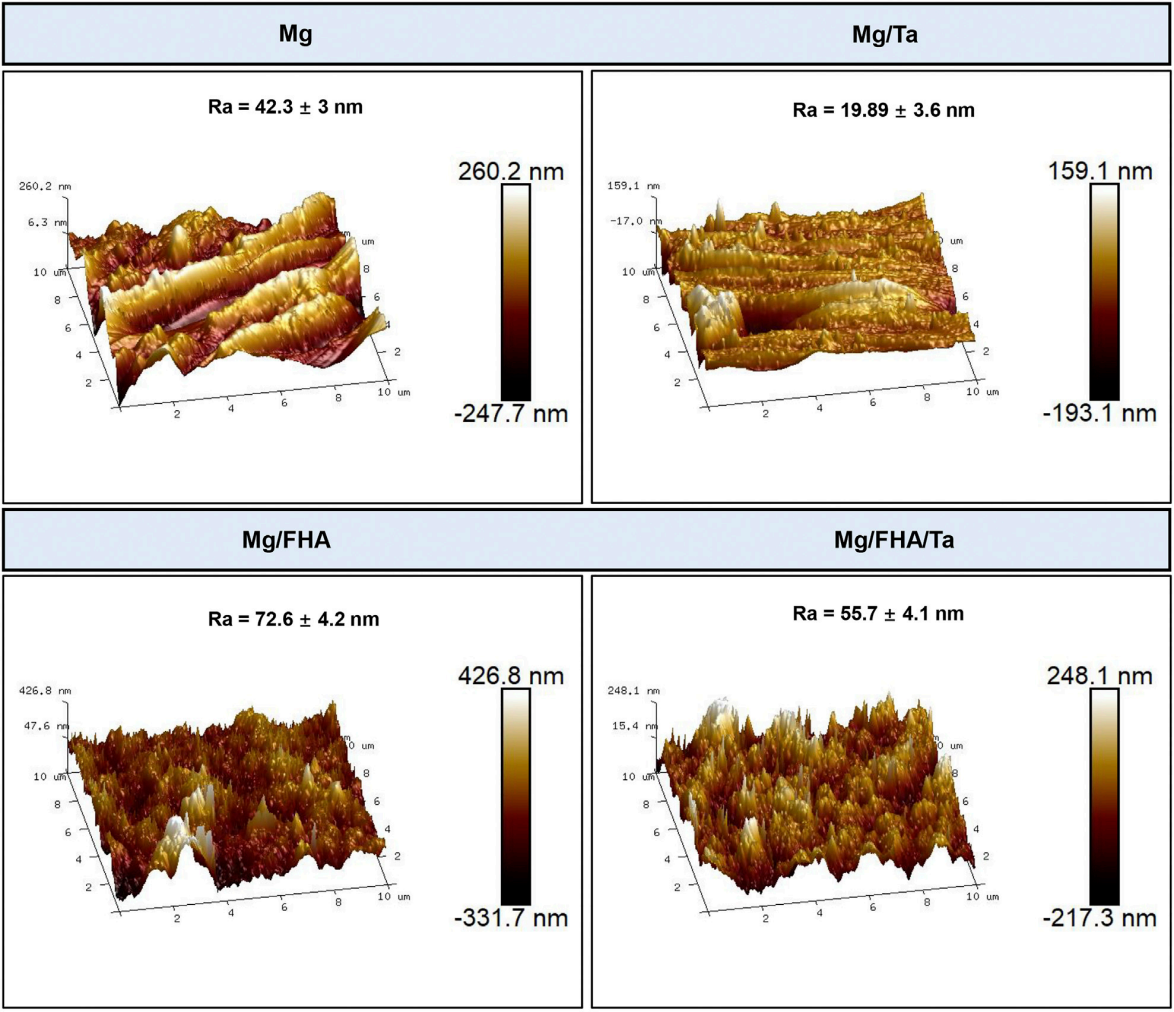
AFM images of different samples and corresponding Ra values (the data is displayed as mean ± standard deviation) (*n* = 6).

The SEM analysis of the samples is depicted in Figure 2. As observed, several scratches were present on the Mg and Mg/Ta substrates, owing to the mechanical polishing (Yu et al., 2018). Further, in contrast with the pure Mg surface, the Ta coated Mg samples consisted of nanoparticles. Moreover, the FHA modified samples were significantly different from the Mg and Mg/Ta samples and revealed a nano-needle-like structure. In the Mg/FHA/Ta group, the nano-needle-like structure was noted to be thick, and the layer consisted of nanoparticles which had been confirmed by previous studies (Tang et al., 2013; Lu et al., 2019; Xu et al., 2019). In addition, the diameter of the needles on the surface and bottom layers was 20 and 40 nm respectively, observed by the SEM images. The FHA nano-needles exhibited a high aspect ratio and similarity with the collagen fibrils, especially the sharp tips. Compared with the pure FHA nano-needles, Mg/FHA/Ta demonstrated a thick nanorod structure. The AFM images in Figure 3 represent the 3D morphology of the different specimens, corresponding to the SEM observation. The Ra value, representing the surface roughness of Mg/FHA (72.6 ± 4.2 nm), was noted to be significantly higher than Mg (42.3 ± 3.0 nm). However, the deposition of Ta decreased the Ra value for the Ta-coated samples, with Mg/FHA/Ta and Mg/Ta exhibiting the values of 55.7 ± 4.1 and 19.89 ± 3.6 nm respectively. The results indicated that the nano-needle structure could significantly enhance surface roughness, while the Ta coating decreased the surface roughness. The observed phenomenon may be attributed to the nano-needle-like structure possessing high surface roughness, whereas the Ta nano-coating enabled the surface to become smooth (Noumbissi et al., 2019). The result corresponds with a previous study which showed that a strontium nanolayer slightly decreased the surface roughness of hot-alkaline treated titanium surfaces (Wang et al., 2020).

**FIGURE 4:**
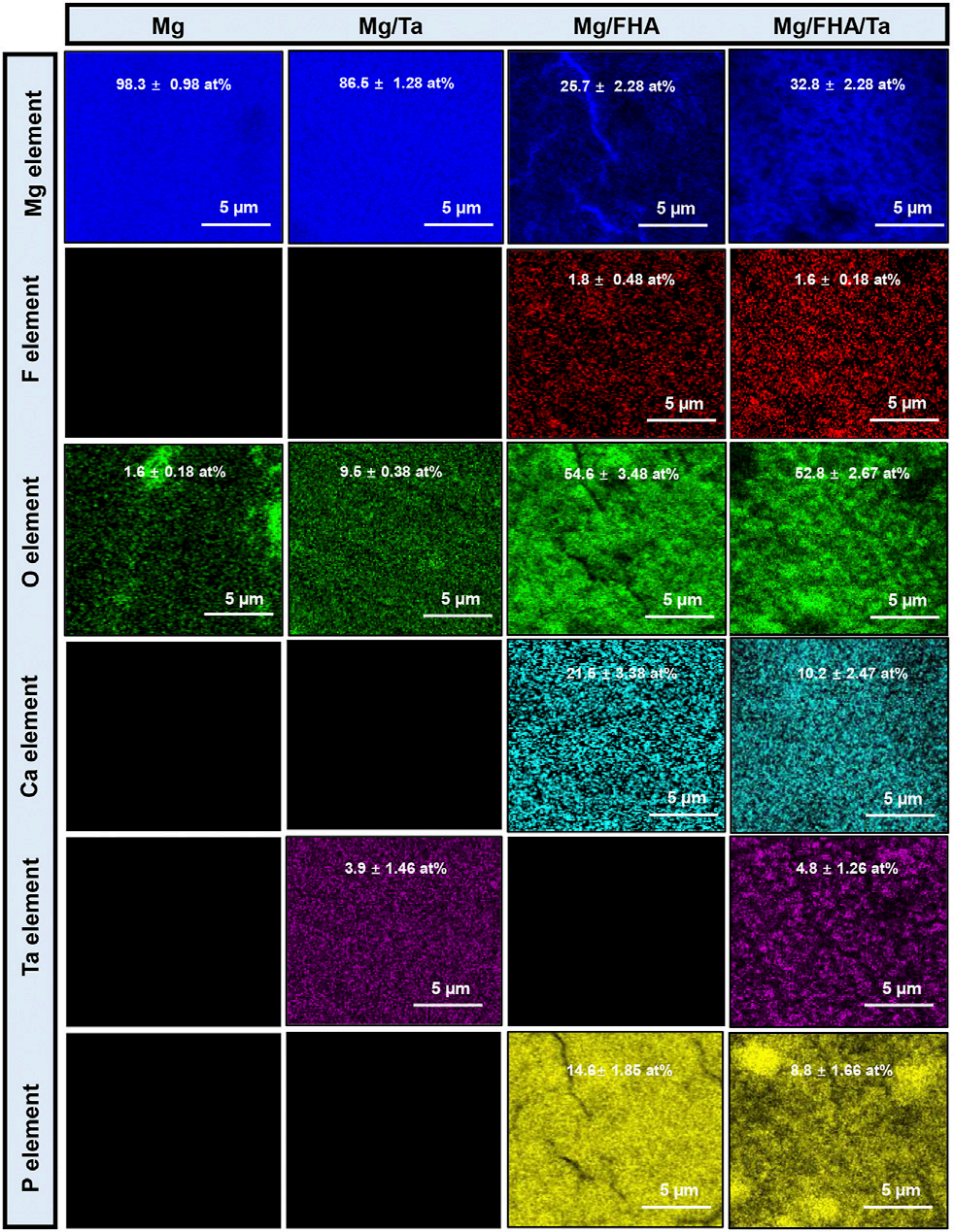
EDS mapping of different samples showing the distribution of the elements Mg, F, O, Ca, P, and Ta (data is displayed as mean ± standard deviation) (*n* = 6).

As shown in Figure 4, the EDS mapping was performed to analyze the elemental distribution maps on the sample surfaces. Only the elements Mg (98.3 ± 0.98 at%) and O (1.6 ± 0.18 at%) were observed on the Mg surface. Moreover, O element (54.6 ± 3.48 at% for Mg/FHA and 54.6 ± 2.67 at% for Mg/FHA/Ta) was the major component in the FHA modified samples. In addition, the content of the Ca element was 21.5 ± 3.38 at% and 10.2 ± 2.47 at% for Mg/FHA and Mg/FHA/Ta, respectively. P element (14.8 ± 4.86 at% for Mg/FHA and 8.8 ± 1.66 at% for Mg/FHA/Ta) plays an important part in the Ca/P ratio, which influences osteogenic properties significantly. The closer the Ca/P ratio was to 1.7, the greater the osteogenic properties it possessed. For Mg/Ta and Mg/FHA/Ta, the content of the Ta element was 3.9 ± 1.46 at% and 4.8 ± 1.26 at%, which indicated the successful deposition of a Ta nanolayer on the Mg and Mg/FHA substrates.

**FIGURE 5:**
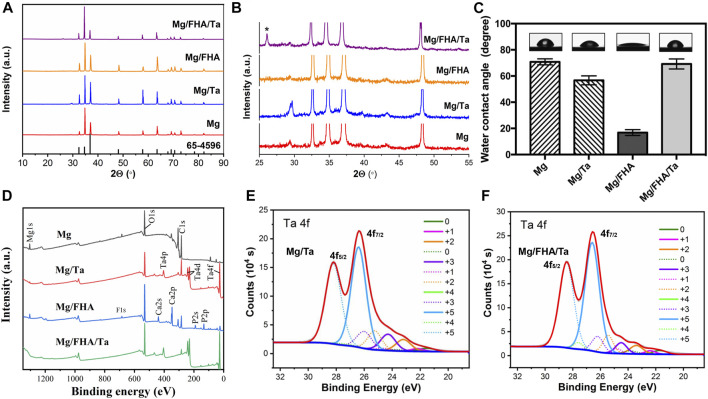
XRD patterns **(A)**, local enlarge XRD patterns **(B)**, water contact angle **(C)** and XPS results of different samples, **(D)** Full spectrum, **(E)** Mg/Ta detail spectrum, **(F)** Mg/FHA/Ta detail spectrum (*n* = 6).

The XRD patterns of the developed samples are presented in Figure 5A. The sharp and intense peak at 25.9° in Mg/FHA/Ta indicated the growth of the deposited FHA crystals, as marked in Figure 5B. Such growth was not significant in Mg/FHA, compared to the Mg/FHA/Ta group. In Mg/FHA group, the brushite (CaHPO_4_.2H_2_O) diffraction peak was not stable, which indicated that the crystallinity and coating thickness were not sufficient to be detected (Zhao et al., 2016; Noumbissi et al., 2019; Wang et al., 2020). This result corresponded with previous studies which found that insufficient coating thickness and crystallinity significantly influenced the XRD detection (Bir et al., 2015). In addition, any changes in the diffraction peaks and crystallinity were not observed on incorporating Ta in the FHA coatings, as compared with the Mg/FHA sample. The results indicated that the Ta coating might be too thin to be detected, or the magnetron sputtering process did not generate the crystal phase of Ta (Wang et al., 2020).

Wettability is an important surface characteristic of implants due to the fact that high hydrophilicity promotes protein adsorption and cell adhesion, thus accelerating osteointegration (Gittens et al., 2014). Water contact angle is usually used to assess the wettability of the material surface. The WCA findings (Figure 5B) revealed no significant difference among the contact angles of Mg (70.8 ± 2.3°), Mg/Ta (56.7 ± 3.4°), and Mg/FHA/Ta (69.2 ± 3.8°). Notwithstanding, Mg/FHA was revealed to possess the highest hydrophilicity (16.8 ± 2.2°), which might be attributed to the superior hydrophilicity of the nano-needle layer (Dorozhkin, 2012).

Moreover, the XPS analysis was also used to characterize the elemental composition and gain insights about the bonding compositions as well as the oxidation state of Ta. The electronic orbitals of Ca 2p, P 2p, and O 1s were observed in the FHA coated samples, corresponding to the binding energies of 348.2, 134.9, and 530.5 eV, respectively. The XPS results in Figure 5D, also revealed that the Ta_2_O_5_state was predominant in Mg/Ta and Mg/FHA/Ta. The detailed scan data of Ta_2_O_5_ XPS revealed that the Ta element was composed of Ta_2_O_5_ (+5), suboxide tantalum (+1 to +4), and Ta metal (0), and that the detected Ta_2_O_5_ could mainly be attributed to the oxidization of Ta in air, which corresponded with the previous studies (Bakri et al., 2019; Zhang et al., 2019; Ding et al., 2021). However, no F1s were detected in Mg/FHA/Ta, while F1s existed in Mg/FHA and Mg with a binding energy of 690 eV. The observed phenomenon might be attributed to the influence of Ta coating and the detection of F1s that existed in Mg was attributed to pollution, and no F was detected after narrow-spectrum splitting.

### Cell Biological Behaviors

**FIGURE 6:**
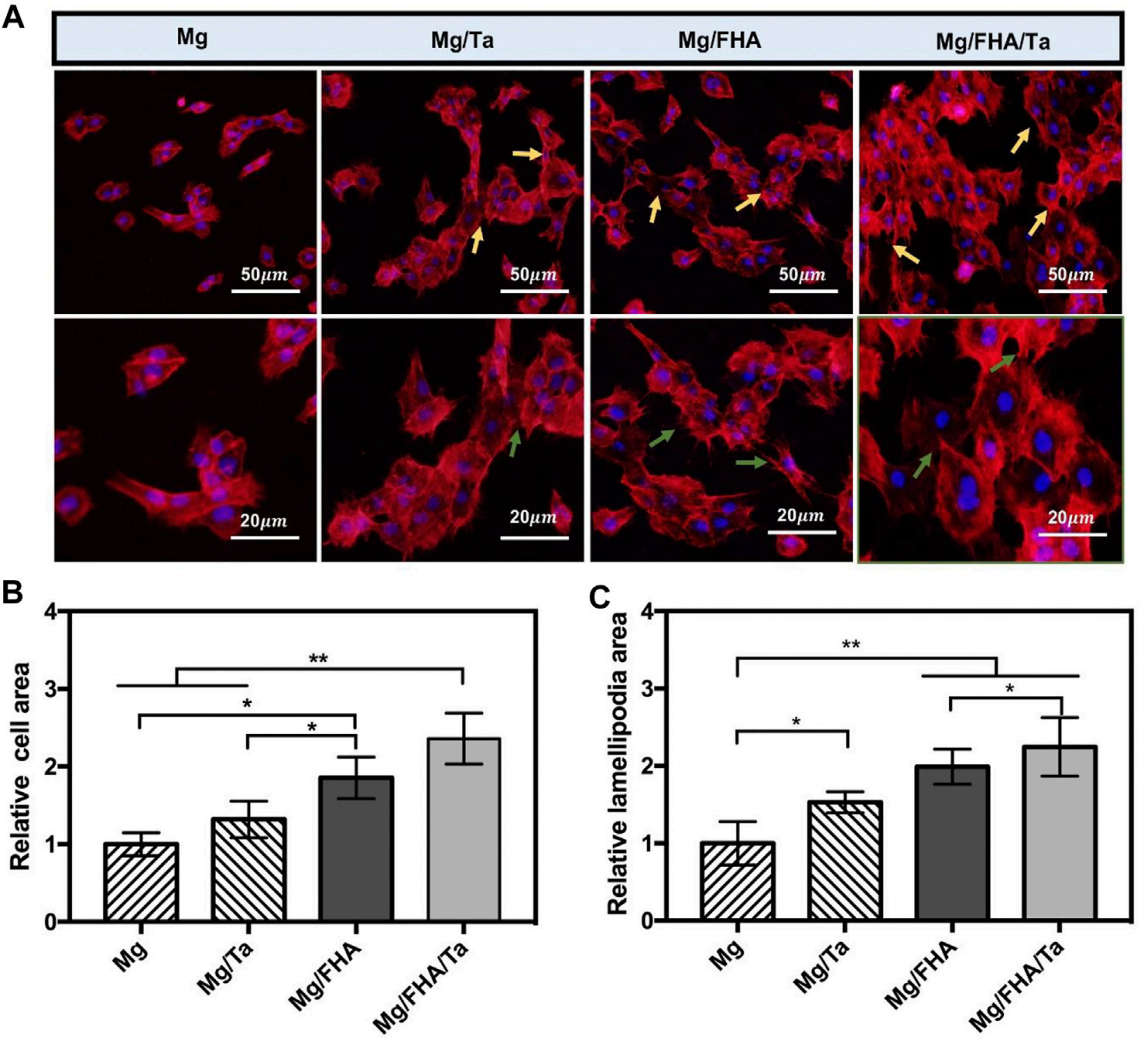
**(A)** Fluorescent images of MC3T3-E1 cells on different surfaces (blue: nucleus, red: cytoskeleton). Spindle-shaped cells were indicated by gold arrows and lamellipodia were indicated by green arrows; Statistics of relative cell areas **(B)** and lamellipodia areas **(C)** according to the fluorescent images, Mg group was taken as a control group, (data displayed as mean ± standard deviation, *p < 0.05, **p < 0.01.) (*n* = 6).

The cell morphology is depicted in Figure 6A after culturing cells on different specimens for 3 days. The MC3T3-E1 cells spread on the Mg/FHA/Ta groups exhibited the largest number with significant extension, while the cells on Mg and Mg/FHA were slender and fusiform. The cells spread on the Mg samples were especially low in number and exhibited a shrunken morphology. The observed phenomenon was mainly attributed to the excess Mg^2+^, which increased the pH value of the cell condition and impaired cell growth (Zaatreh et al., 2017). The well-elongated osteoblasts were beneficial for the osteogenic differentiation, thus, facilitating cell-cell communication to coordinate the cell behavior (Sun et al., 2018). Moreover, the MC3T3-E1 cells stretched out with more lamellipodia in the case of Mg/FHA/Ta groups (marked with blue arrows) as compared with Mg and Mg/Ta, which are considered to be essential for cell migration and cell adhesion to implants as anchor points (Ho et al., 2014). The quantitative analyses based on the relative cell and lamellipodia areas, both Mg/FHA and Mg/FHA/Ta groups exhibited the optimal cell spreading and lamellipodia formation, followed by Mg/Ta (Figures 6B,C). The surface topological nanoscale structure has a vital effect on the adhesion, migration, and morphology of osteoblasts (Gittens et al., 2011; Puckett et al., 2008; Khrunyk et al., 2020; Yeo, 2019). In addition, the Ta ion function has also been proven to enhance the growth and differentiation of osteoblasts, which combined with the nanostructure synergistically enhances the cellular responses (Wang et al., 2018). A previous study has demonstrated that the nanostructured surfaces exhibited superior hydrophilicity and excellent protein adsorption behavior, thus, the well-elongated MC3T3-E1 cells with mature and thick pseudopodia could be observed on the Mg/FHA and Mg/FHA/Ta surface (Li et al., 2015). Although the MC3T3-E1 cells were sensitive to the ion concentration, a large content of Mg ions from Mg and Mg/Ta impaired the cell spreading, as compared with Mg/FHA and Mg/FHA/Ta. However, an appropriate amount of Ta and Mg ions enhanced the attachment and spreading of osteoblasts. The earlier literature study has proved that the cell behavior of osteoblasts incubated on Ta metal-containing substrates is evident from obvious stress fibers and actin filaments (Zhang et al., 2021). Similarly, both Mg/FHA and Mg/FHA/Ta exhibited well-elongated MC3T3-E1 cells in this study. In addition, the results corresponded with a previous study showing that nanoscale Ta morphology promoted cell behavior more than a smooth surface, and that bi-layer architecture possessed the best biological properties for this (Zhang et al., 2021; Vlacic-Zischke et al., 2011).

**FIGURE 7:**
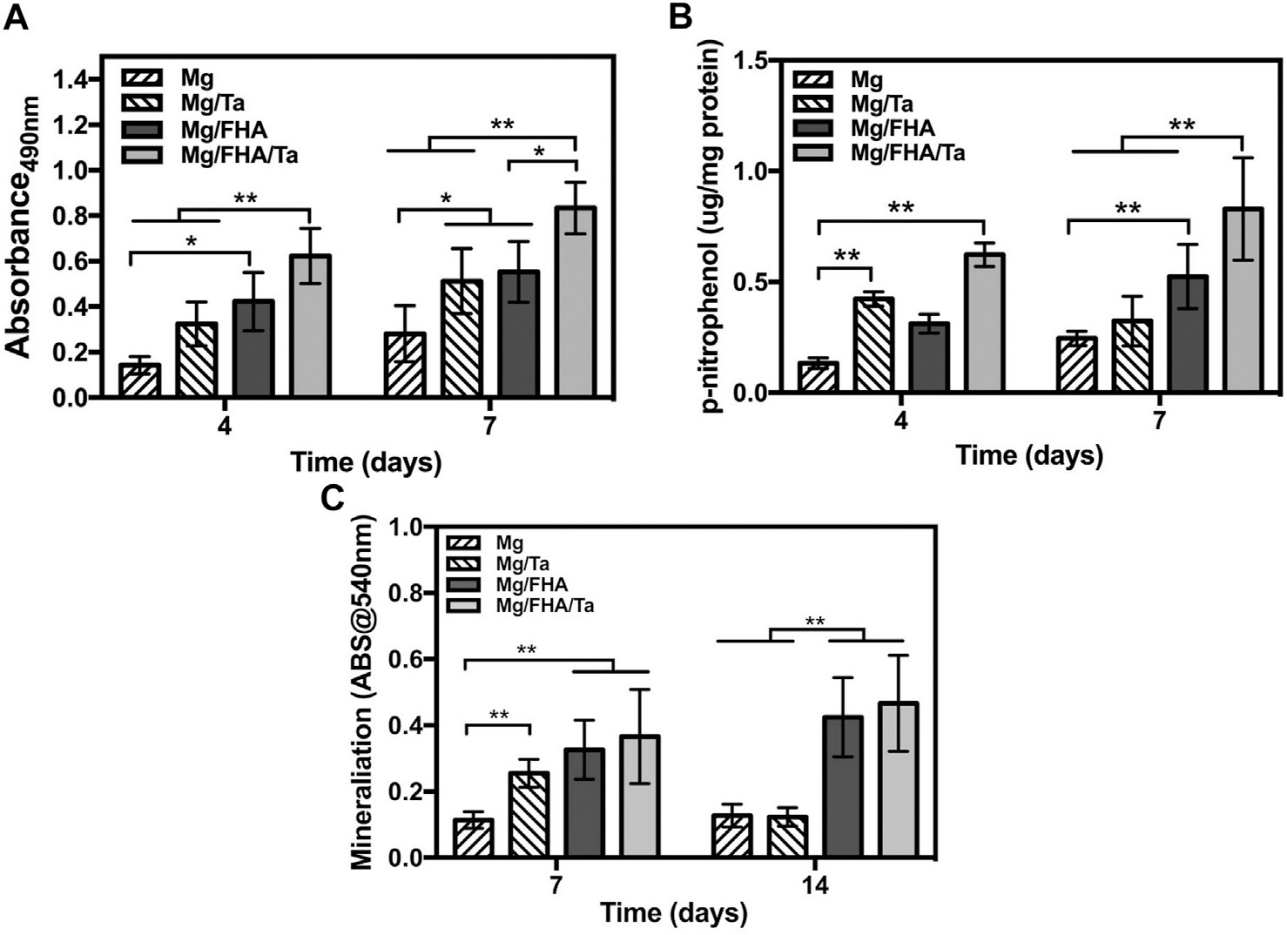
Cell viability **(A)** and ALP activity **(B)** of MC3T3-E1 cells on 4 and 7 days; **(C)** quantitative analysis of mineralization after 7 and 14 days, (the data was displayed as mean ± standard deviation, *p < 0.05, **p < 0.01.) (*n* = 6).

The cell viability of the MC3T3-E1 cells on the samples was revealed by MTT evaluation. As shown in Figure 7A, the cell viability on Mg/FHA/Ta was superior to the other groups after 4 days (especially Mg and Mg/Ta, *p* < 0.01). Further, the same tendency was observed at 7 days (*p* < 0.01), which was consistent with the previous findings that the FHA and Ta coatings promoted the cell viability of osteoblasts (Wang et al., 2018; He et al., 2014). Moreover, the combination of FHA coating and Ta played a synergistic role in promoting cytocompatibility. The cell viability assay indicated that the FHA coating played a more effective role in promoting the cytocompatibility as compared to the Ta coating, which mainly attributed to the thicker layer of FHA coating and the beneficial nano-needle like structure. The result corresponded to a previous study suggesting that FHA coating possessed excellent biocompatibility (Yu et al., 2018; Wang et al., 2016). What is more, the surface roughness and hydrophilic properties could regulate osteoblast behavior, and the higher surface roughness and hydrophilicity may benefit osteoblast growth (Wang et al., 2016; Wan et al., 2016; Huang et al., 2016). According to the AFM and WCA results, Mg/FHA possessed the highest surface roughness and hydrophilicity. Therefore, it is another factor that Mg/FHA showed better than Mg/Ta. In addition, though Mg/FHA/Ta showed comparatively low surface roughness and hydrophobicity, it possessed the best cell growth, which we mainly attributed to the synergistic effects of Ta function and a bio-mimicing surface.

The early osteogenic differentiation and late extracellular matrix mineralization properties of the MC3T3-E1 cells were determined by ALP activity and alizarin red staining analyses, respectively. As shown in Figure 7B, the ALP activities of the MC3T3-E1 cells on Mg/Ta, Mg/FHA, and Mg/FHA/Ta were considerably higher than Mg after 4ays and 7 days (*p* < 0.01), while Mg/FHA and Mg/Ta exhibited no statistically significant difference. Moreover, the ALP activity of Mg/FHA/Ta was significantly higher than Mg, Mg/Ta, and Mg/FHA after 7 days (*p* < 0.01). It indicated that Mg/FHA possessed a superior ALP activity than Mg/Ta, and Mg/FHA/Ta exhibited the best ALP activity, mainly attributed to the thicker FHA coating than the tantalum coating, thus, preventing the Mg degradation more effectively. From a bio-mimicry point of view, both the Mg/FHA/Ta and Mg/FHA possessed the bone bionic surface (the nano-needle or nano-rod like structure proved by SEM observation) which provide beneficial conditions for cell differentiation (Wan et al., 2016; Huang et al., 2016). Therefore, the more cell activity was provided by the substrate, the better the ALP activity. The extra-mineralization analysis in Figure 7C revealed a higher OD 540 nm value of Mg/FHA/Ta and Mg/FHA than Mg after 7 days of osteoinduction (*p* < 0.01). Moreover, Mg/FHA/Ta and Mg/FHA possessed significantly higher optical density than Mg and Mg/Ta after 14 days of osteogenic induction. It indicated that Mg/FHA/Ta and Mg/FHA possessed superior late extracellular matrix mineralization behavior and favorable early osteogenic differentiation characteristics. Further, compared with 7 days, the OD540 nm value of Mg/Ta decreased after 14 days of osteogenic induction, which might be attributed to the degradation of the Ta coating and excess release of Mg ions, thus, decreasing the cell viability and impairing the mineralization level (Chen et al., 2018). Compared with Mg/Ta, Mg/FHA exhibited superior hydrophilicity and bioactivity. Therefore, as compared to Mg/Ta, Mg/FHA effectively promoted early ALP activity and mineralization levels in osteoblasts, attributed to the thicker coating layer and better biocompatibility. Generally, Ta has been confirmed to be involved in increasing COL-1, mineralization and ALP activity (Liu et al., 2016). Overall, the combination of the FHA and Ta coatings enhances the osteogenic property of the surface. Based on the cell viability assay, the FHA coating provided a more advantageous condition for cell viability as compared to the Ta coating. However, the Ta coating seemingly enhanced the ALP activity to a larger extent as compared with the FHA coating during the first 4 days, which indicated that the Ta function might have played a pivotal role in osteogenic induction. In addition, the comparatively stable FHA coating endowed the surface with a non-toxic character, thus, the ALP activity of Mg/FHA and Mg/FHA/Ta increased more effectively 7 days after osteoinduction. Moreover, the mineralization analysis revealed a similar trend. Thus, the Ta coating played a more important role in promoting osteogenic properties, and the FHA coating had a significant role in enhancing cytocompatibility. In addition, studies have proven that the nanostructure surface also plays an important role in osteogenic promotion (Ding et al., 2019; Lee et al., 2015). Thus, the combination of the FHA and Ta coatings could synergistically enhance the biological property of the samples. In order to further evaluate the biological function of the developed implants, *in vivo* experimental analysis and mechanism identification will be performed subsequently for optimization.

## Conclusion

The Mg/FHA/Ta surface was successfully fabricated by hydrothermal synthesis and magnetron sputtering. The FHA coating endowed the surface with a nano-needle structure, along with enhancing surface roughness and hydrophilicity and altering surface elemental distribution. The Ta coating allowed the formation of a thin nano-layer on the surface, thus decreasing surface roughness and hydrophilicity. Mg/FHA/Ta was confirmed to possess optimal cell viability, cell spread behavior, and osteogenic differentiation, which were mainly attributed to the stable FHA layer and long-lasting Ta ion release. The *in vitro* biological analysis proved that the FHA coating imparted the surface with cell viability and osteogenic property, and the Ta ions played a pivotal role in osteogenic differentiation. The combination of FHA and Ta coatings could synergistically promote the biological function, thus, providing a novel idea for surface modification.

## Data Availability

The original contributions presented in the study are included in the article/Supplementary Material, further inquiries can be directed to the corresponding authors.
